# Topical Anti-Inflammatory Therapies in Veterinary Medicine: Advancing Animal Health Through a One Health Approach

**DOI:** 10.3390/ani16081252

**Published:** 2026-04-18

**Authors:** Maria-Teodora Pițuru, Miruna-Maria Apetroaei-Leucă, Gabriela Ștefan, Cosmin Șonea, Dana Tăpăloagă, Bruno Ștefan Velescu, Andreea Letiția Arsene, Denisa Ioana Udeanu, Marina Ionela Nedea, Constantin Vlăgioiu

**Affiliations:** 1 Faculty of Veterinary Medicine, University of Agronomic Sciences and Veterinary Medicine of Bucharest, 105 Splaiul Independentei, 050097 Bucharest, Romania; maria.pituru@fmvb.usamv.ro (M.-T.P.); cosmin.sonea@fmvb.usamv.ro (C.Ș.); dana.tapaloaga@fmvb.usamv.ro (D.T.); constantin.vlagioiu@fmvb.usamv.ro (C.V.); 2 Faculty of Pharmacy, “Carol Davila” University of Medicine and Pharmacy, 6 Traian Vuia Street, 020956 Bucharest, Romania; gabriela.stefan@drd.umfcd.ro (G.Ș.); bruno.velescu@umfcd.ro (B.Ș.V.); andreea.arsene@umfcd.ro (A.L.A.); denisa.udeanu@umfcd.ro (D.I.U.); marina.nedea@umfcd.ro (M.I.N.)

**Keywords:** One Health, topical anti-inflammatory therapy, veterinary dermatopharmacology, environmental risk assessment, drug residues in food-producing animals, sustainable veterinary formulations, translational veterinary pharmacology

## Abstract

This narrative review examines topical anti-inflammatory therapies in veterinary medicine through the lens of the One Health framework, integrating pharmacology, dermatology, ecotoxicology, food safety, and regulatory science. It discusses the interconnected roles of veterinarians, pharmacists, environmental scientists, public health authorities, and regulatory bodies in addressing antimicrobial resistance, environmental contamination, zoonotic transmission, and drug residues in food-producing animals. By emphasising cross-sector collaboration, the review highlights how coordinated strategies can enhance animal welfare, safeguard human health, and reduce ecological burden. The article analyses inflammatory conditions in companion and farm animals and compares systemic versus topical anti-inflammatory approaches. Particular attention is given to corticosteroids, NSAIDs, immunomodulators, pro-resolving lipid mediators, and plant-derived bioactives, alongside advances in vehicles such as lipid nanocarriers and biodegradable film-forming systems designed to minimise systemic absorption and environmental dispersion. Regulatory considerations, residue control, pharmacovigilance gaps, and sustainability-oriented formulation strategies are critically addressed. Topical anti-inflammatory therapies, when rationally designed and monitored under One Health principles, represent a strategic opportunity to improve therapeutic precision while limiting systemic toxicity and ecological impact. Future directions should prioritise translational research, eco-compatible formulation design, and harmonised regulatory frameworks.

## Simple Summary

Inflammation is one of the most common health problems in animals, and treating it effectively while minimising harm to humans, animals, and the environment is a growing priority in modern veterinary medicine. This review examines medicines applied directly to the skin or body surface of animals, known as topical anti-inflammatory treatments, and evaluates their advantages over medicines given orally or by injection. When drugs are applied locally, they act where they are needed most, reducing unwanted side effects and the risk of harmful residues entering the food chain or the environment. The review discusses which skin conditions in companion animals and farm animals benefit most from this approach, compares the main drug classes available, and analyses the newest formulation technologies designed to make these medicines safer, more effective, and more environmentally friendly. The importance of collaboration between veterinarians, pharmacists, public health professionals, and environmental scientists in developing and regulating these treatments is highlighted. Responsible use of topical veterinary medicines represents a practical strategy to improve animal welfare, protect human health, and reduce environmental pollution simultaneously.

## 1. Introduction

Inflammation and pain are the most prevalent and predominant symptoms of numerous illnesses. Inflammation, the most critical response to tissue injury, generates a range of adverse stimuli, including infections, noxious chemical compounds, and physical damage, which result in redness, oedema, heat, and pain [1,2].

Topical anti-inflammatory treatments are pharmaceutical forms administered directly to the skin, mucous membranes, or external epithelium to reduce local inflammation [3]. Depending on the underlying pathology, these preparations can be formulated in various forms or combined with other active substances, such as multiple antimicrobials, antihistamines, and antipruritics. Among the most commonly formulated anti-inflammatory active substances are corticosteroids, non-steroidal anti-inflammatory drugs (NSAIDs), immunomodulatory agents, and various active ingredients of natural origin, which can be formulated as lotions, gels, ointments, sprays, foams, or transdermal patches [4,5]. These are commonly used in both pets and farm animals to treat allergic dermatitis, otitis externa, localised trauma, reactions to insect bites, surgical wounds, and mastitis.

In veterinary medicine, there is a growing demand for various topical pharmaceutical forms, in line with a broader trend toward localised, precision-based therapy models that limit systemic exposure while achieving personalised therapeutic outcomes [6,7].

The World Health Organisation (WHO) defines the concept of “One Health” as a set of principles that supports the interconnectedness of human, animal, and environmental health [8]. Thus, through collaboration between these sectors and professionals, the aim is to use transdisciplinary methods to manage health problems affecting multiple species, particularly those related to antimicrobial resistance, zoonotic infections, and environmental pollution [9].

In this context, the One Health concept has the potential to revolutionise multiple sectors through multidisciplinary collaboration [10]. One such example is the development of new pharmaceutical formulations for veterinary medicine. Medicines administered to animals can directly and indirectly influence ecosystems, the microorganisms present in them, animal workers, food chains, and the health of people who consume animal products [11]. An important example in this regard is antiparasitic and anti-inflammatory drugs used on animal skin, which can enter aquatic habitats through runoff or disposal and, over time, contribute to the development of antimicrobial resistance and increased pollution [12,13]. Therefore, during the development, regulation, and use of veterinary treatments, it is necessary to assess both their effectiveness in treating the disease and their overall impact on the environment and humans.

It is important to note, however, that topical pharmaceutical forms offer several advantages over systemic anti-inflammatory drugs. Firstly, topical preparations enable drugs to be administered directly to the site of inflammation, which maintains high drug levels in that area while avoiding first-pass metabolism and reducing systemic absorption [14]. Second, less systemic exposure means fewer side effects, such as immunosuppression from chronic corticosteroid therapy, gastrointestinal ulcers, and kidney damage from chronic therapy or high-dose NSAIDs, which are common with systemic treatments [15]. Thirdly, topical administration is often easier to achieve in animals when oral or injectable administration is not possible due to behaviour, stress, or body shape (e.g., horse limbs, dog ears, and cow udders) [16].

Even with these benefits, there remain multiple challenges in developing pharmaceutical formulations useful in veterinary medicine while adhering to the principles of One Health. Regulatory differences, the lack of common efficacy criteria, the limited number of clinical studies specific to veterinary medicine, and the inconsistent quality of products are the main limitations to the full integration of topical anti-inflammatory drugs into routine veterinary pharmacotherapy. In addition, concerns about environmental residues, non-compliant use, and still underdeveloped pharmacovigilance systems increase the importance and need to develop new therapies in line with One Health guidelines [17].

This narrative review critically analyses topical anti-inflammatory therapies in veterinary medicine within the framework of the One Health concept, integrating pharmacological, clinical, environmental, and regulatory perspectives. Specifically, the review seeks to (i) examine the pathophysiology of inflammatory conditions in companion and farm animals that require topical management; (ii) compare systemic and topical anti-inflammatory strategies in terms of efficacy, safety, residue risk, and environmental impact; (iii) evaluate currently marketed products and emerging active pharmaceutical ingredients and formulation technologies; and (iv) highlight the role of interdisciplinary collaboration among veterinarians, pharmacists, environmental scientists, and regulatory authorities in optimising therapeutic outcomes while safeguarding animal welfare, public health, and ecosystem sustainability.

## 2. The One Health Framework and Its Relevance to Veterinary Pharmaceuticals

### 2.1. The One Health Concept: A Brief Overview

**Figure 1:**
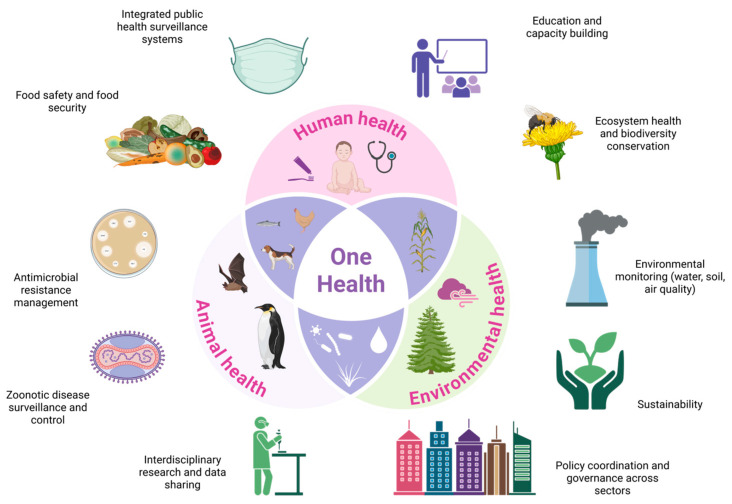
One Health pillars (Created with Biorender.com).

The One Health initiative is a collection of strategies that have been developed to establish enforcement programmes, laws, regulations, and research across multiple sectors, aiming to enhance the outcomes of public health programmes. An approach such as this is necessary to mitigate potential risks to public health that arise at the intersection of animals, people, and the environment. Zoonosis control, laboratory testing, tropical infectious illnesses, antibiotic resistance, and the preservation of plants and the environment are among the areas of interest intended to be implemented in accordance with the One Health concept [18,19,20]. This research collection of initiatives provides the opportunity to achieve a comprehensive understanding of a variety of health solutions and to gain a complete picture of the connections between the health of plants, animals, and their surroundings and their impact on human health. It is possible to uncover highly significant yet commonly overlooked substances by examining the most widespread problems from the perspective of the entire human environment (Figure 1). This variety of activities may thus lead to the development of intervention techniques that are more informed on the subject [21,22].

When one considers the multitude of facets and the specific characteristics of each public health problem, it becomes abundantly clear that the integrated One Health strategy cannot be disconnected from the concept of “ecological or sustainable health”. The presumption is that, in a polluted world characterised by socioeconomic and political instability, as well as the loss of natural resources, it may become challenging to protect the health and well-being of people [10,23,24]. In addition, the implementation of a One Health approach would create a scientific environment that would not only allow for the sharing technological resources, for instance workspace or laboratory resources, among a wide variety of experts, such as physicians, chemical engineers, veterinarians, and microbiologists, but would also allow for a greater possibility of sharing resources within the academic community [25]. Unfortunately, biological, wastewater, and applicable chemical samples may be difficult to obtain for financial or ethical reasons. With the principle of One Health, on the other hand, these samples may be shared among several research groups with diverse interests in infections and toxic substances, thereby enabling the development of diverse views and methods [19,26].

The rising influence of zoonotic infections on the well-being of people and other animals has been brought to the attention of the general public once again, as a result of recent disease outbreaks worldwide, such as COVID-19, which is a concern related to the One Health concept [27,28]. In addition, it is becoming increasingly clear that zoonotic disease epidemics are caused by a range of factors, including human interference with habitats that support wildlife, climate change, agricultural intensification, and the rise in the world population. In addition, the contamination of food products with poisonous compounds and other pests affects the human population of the planet [29,30]. It is generally acknowledged that One Health is a fundamental pillar of the measures used to manage and prevent diseases on a global scale [31].

At the macro level, the most significant players engaging in One Health include international organisations, national governments and ministries, universities, non-profit organisations, academics, and local communities [32]. On the other hand, research organisations are active as well. The management of public health concerns and the implementation of One Health programmes are essentially the result of collaboration among all these stakeholders at multiple levels of authority [33].

### 2.2. Topical Veterinary Treatments Within the One Health Framework

It should be noted that the One Health initiative emphasises the importance of developing new strategies to improve human and animal health outcomes while preserving the environment. In this context, through continuous collaboration between pharmacists, veterinarians, agri-food professionals, and environmental protection professionals, research in the field of pharmaceutical development for veterinary medicine applications can be advanced [34]. At the same time, in the context of the need to protect animal health, the development of new pharmaceutical preparations can have a number of advantages that can become a solution for several directions set out in One Health.

Firstly, veterinary medicines, especially topical pharmaceuticals, can reduce the risk of zoonotic infection transmission [35,36]. This is because 60–75% of new infectious diseases in humans originate in animals [37]. On one hand, this can be achieved by effectively controlling cutaneous infections caused by pathogens with zoonotic potential, such as *Staphylococcus pseudintermedius*, *Microsporum canis*, and *Leishmania* spp. Topical therapy reduces the microbial burden at the skin surface and limits the likelihood of direct transmission to humans through contact with infected animals [38]. On the other hand, by substituting or reducing the need for systemic antimicrobial treatment, targeted topical therapy decreases the overall selective pressure for antimicrobial resistance [39], which represents one of the most critical threats at the human–animal-environment interface under the One Health framework. *Staphylococcus pseudintermedius*, for example, is a common commensal and opportunistic pathogen in dogs. Its genetic characteristics confer increased resistance, similar to those of *Staphylococcus aureus*, allowing it to spread to other animals through direct contact [40]. *S. pseudintermedius* is recognised as a resident of the skin and mucous membranes, constituting part of the normal microbiota in dogs. This pathogen is acknowledged as opportunistic and zoonotic, capable of colonising humans and inducing severe diseases, particularly in immunocompromised individuals [41]. Methicillin-resistant *S. pseudintermedius* (MRSP), characterised by intrinsic multidrug resistance, has emerged as a significant public health concern. The epidemiological situation is worsened by reports of zoonotic transmission and human infections, primarily linked to the rising prevalence of dog ownership and the close interactions between dogs and humans [42,43]. Evidence regarding the zoonotic transmission of MRSP from pet dogs to humans, including dog owners, small-animal veterinarians, and individuals in close proximity to dogs, is limited, particularly due to inaccurate identification of *S. pseudintermedius* as *S. aureus*. Reports on the rising emergence and dissemination of MRSP in humans have steadily increased since its initial documentation in Belgium in 2006 [41]. The emergence of MRSP strains has compromised treatment outcomes in veterinary and human medicine, as these strains exhibit resistance to β-lactam antimicrobials, which are typically prescribed as first-line treatments [41,43]. The inadequate understanding and monitoring of the zoonotic transmission of *S. pseudintermedius* have led to an underestimation of its transmission extent, incidence, epidemiology, and public health importance [41]. Similarly, topical therapies for *Leishmania* spp., a protozoan parasite that can infect both dogs and humans, can also break transmission cycles, especially in endemic areas where infections with this parasite are prevalent [44,45]. Thus, targeted, safe, and species-specific therapies used in veterinary clinical practice could considerably reduce the risk of spreading infections to humans [46]. In this context, as animal reservoirs are of utmost importance for controlling human infectious diseases, the development and regulation of topical veterinary products with zoonotic effects must align with the principles of One Health. The frequent or inappropriate use of antimicrobials in veterinary medicine contributes to the increased risk of developing antimicrobial resistance, an alarming global public health problem affecting humans, animals, and the environment [47,48]. Among the main common errors contributing to the increase in antimicrobial resistance are, but are not limited to, the empirical use of antimicrobials without confirmation of infection, misidentification of the causative agent and consequent administration of the wrong class of antimicrobials (the most common example being the prescription of an antibiotic for viral or parasitic infections) or the use of antimicrobials with an unjustifiably broad spectrum [49,50]. Although topical antibiotics are often considered to be low risk, their use may nevertheless favour the selection of resistant bacterial strains, such as MRSP and extended-spectrum β-lactamase-producing members of the *Enterobacteriaceae* family [38]. These strains can persist on the skin of animals, in the domestic environment, and in wastewater. The Committee for Medicinal Products for Veterinary Use strategy on antimicrobials 2021–2025 emphasises that drug resistance in animal pathogens can negatively influence the effectiveness of treatments in humans, particularly in contexts of close contact, cohabitation, or environmental contamination. Incorrect use of topical antibiotics, in the absence of professional supervision or in subtherapeutic doses, exerts increased selective pressure without eliminating the etiological agents and favours the emergence and persistence of resistant strains [51]. In this context, the One Health initiative on combating antimicrobial resistance supports the development and rational, evidence-based use of topical veterinary preparations.

On the other hand, topical veterinary medicines can enter the environment through various routes, such as hair loss from animals, contact with soil or sludge, or bathing in surface waters. These chemicals can have a long persistence in water and soil after discharge, with harmful effects on ecosystems and aquatic animals [52,53]. For example, fipronil, imidacloprid, and permethrin, which are chemicals commonly used in spot-on antiparasitic treatments, have been found in surface waters in concentrations that pose a risk to the environment [54,55]. According to the European Medicines Agency, the approval of a medicine must be accompanied by a rigorous environmental risk assessment, particularly for substances with a high potential for dispersion in the environment or long persistence in ecosystems [56]. Topical residues are an underestimated hazard because they are widely used in pets. To address this, we need better disposal procedures, greater public awareness, and the creation of eco-friendly formulations [57,58]. To ensure the health of animals, humans, and the environment, ecotoxicology must be integrated into the development of veterinary pharmaceuticals.

To ensure food safety, topical medications administered to food-producing animals, such as mastitis treatments or antibiotic skin preparations, must comply with stringent regulations on permissible residue levels and withdrawal periods prior to animal consumption. European Union Regulation EC No. 470/2009 establishes maximum residue limits (MRLs) for pharmacologically active chemicals used in animals intended for human consumption [59]. Although the majority of individuals who interact with animals may assume that topical substances do not result in substantial systemic exposure, it is widely recognised by professionals that a compromised skin barrier facilitates the absorption of substances that come into contact with the skin [60,61]. For example, in dairy farm animals requiring udder therapy, residues may enter milk or accumulate in tissues, ultimately posing a risk to human health [62,63]. Therefore, risk assessment techniques must take into account the site of administration, the formulation type, the pharmacokinetic characteristics of the species, and the dynamics of tissue degradation over time. Compliance with these requirements contributes to both the protection of public health and the maintenance of animal products on the market, while supporting the One Health principle of food chain safety.

Although animal welfare is an integral part of the One Health concept, it is not consistently given the attention it warrants. Proper management of pain, inflammation, and dermatological conditions, particularly through the use of easy-to-apply, well-tolerated topical pharmaceuticals, significantly improves animal comfort. In addition, it can lead to improved overall health, productivity, disease resistance, and the ability to interact harmoniously with humans [64,65]. The World Organisation for Animal Health (WOAH) states that improving animal welfare is consistent with public health objectives because it stops the spread of zoonotic diseases, reduces stress-related immunosuppression, and meets society’s standards for ethical animal care [66,67]. For example, animals with untreated skin lesions or long-term inflammatory diseases may be more susceptible to secondary infections that pose important public health risks, such as *Corynebacterium* spp., *Streptococcus* spp., and *Staphylococcus* spp. [68]. The development of veterinary pharmaceuticals must prioritise the creation of safe and effective topical products that safeguard animal welfare and enhance public confidence and sustainability throughout the One Health continuum, as an increasing number of individuals seek animal products that are produced in accordance with the most recent international standards. From a One Health perspective, the failure to adequately manage inflammatory skin conditions in animals carries consequences that extend beyond individual animal welfare. Untreated or poorly managed cutaneous lesions create a favourable environment for secondary colonisation and proliferation of opportunistic bacteria, several of which are recognised as pathogens of relevance to both veterinary and human medicine [61]. In farm animal settings, this increases the risk of transmission to animal handlers and farm workers through direct contact and may necessitate systemic antimicrobial therapy, thereby contributing to the selection of antimicrobial resistance at the human–animal interface. Effective topical anti-inflammatory management, therefore, functions not only as a welfare intervention but as a proactive One Health strategy to reduce infection burden, limit antimicrobial use, and protect the health of all species involved in the care chain.

Nevertheless, topical veterinary medications can have a direct impact on humans, particularly those responsible for animal care, such as veterinarians, farmers, and pet owners. This can occur through skin absorption, aerosolisation during administration, or accidental contact with the mouth. In the Opinion paper released by the European Food Safety Authority (EFSA), a pertinent example was provided to underscore concerns regarding occupational exposure to bupirimate [69]. Depending on the concentration of the molecule and how often it comes into contact with the skin, repeated exposure without appropriate personal protective equipment can cause allergic reactions, endocrine disruption, or toxicological consequences. Similarly, certain components of topical veterinary products lack a secure equivalence in human medicine, thereby making them significantly more harmful in the event of accidental contact [70]. This means that the formula must be carefully designed with human safety in mind, including minimal volatility, skin tolerability, and unambiguous labelling for safety. Adding human risk assessment to the veterinary drug development process is an important step toward making the One Health concept a reality and protecting all species involved in the care chain.

The expansion of global animal commerce and cross-border cattle movement enhances the likelihood of disease dissemination, including zoonoses and resistant organisms. Diseases such as avian influenza, African swine fever, and brucellosis have shown that animal movements can cause large epidemics that affect both public health and the economy [71,72]. The FAO-WHO-WOAH tripartite partnership underscored the importance of developing consistent standards for veterinary medicines, such as those for labelling, safety assessment, and residue management, to help coordinate disease control under the One Health principle [73]. Different countries have different rules on the availability of medicines, quality, and regulatory monitoring, which can make biosecurity measures less effective and hamper the response to outbreaks worldwide. Standardised methods for the approval, monitoring, and use of veterinary medicines are therefore needed to ensure traceability and uniform therapeutic effectiveness, regardless of their origin [74]. The implementation of pharmaceutical governance in accordance with the One Health principle enhances our preparedness, strengthens trade safety, and mitigates the likelihood of emerging health crises at the human–animal-environment interface. In numerous locations, the infrastructure for the systematic collection and analysis of adverse events associated with medication in animals is insufficient, and veterinary pharmacovigilance systems have not yet been completely established. This dearth of surveillance complicates the identification of signs of toxicity, drug-disease interactions, and undesirable effects that could have an impact on the environment and humans [75]. Various studies have underlined that spontaneous reporting rates for veterinary medicines are much lower than for human pharmaceuticals [76]. This is partly because people do not report them as often, are unaware of them, and the digital infrastructure is not sufficiently developed. Poor pharmacovigilance makes it difficult to respond to new safety issues, particularly for medicines applied to the skin or easily released into the environment, which may be dangerous to multiple species. To improve pharmacovigilance networks, veterinary professionals need ongoing training, integrated data systems, and rules that allow veterinarians and animal owners to report problems [76]. Early detection of signals is beneficial for animal welfare, as well as for protecting the environment and humans [77]. Topical veterinary medications may cause ecosystem pollution when utilised without regard for their environmental impact. In this context, topical therapies, formulated and administered according to the One Health strategy, can reduce systemic exposure and support targeted treatment, reducing the overall ecological burden. The integration of life cycle environmental risk assessments into product development and regulatory approval is imperative, and the integration of One Health research directions into current practice could support pharmaceutical innovation with environmental conservation by promoting cross-sectoral responsibility in veterinary medicine, public health, and environmental policy.

Finally, topical veterinary medicines could also offer a range of environmental benefits within the One Health concept, as they leave fewer pharmaceutical residues in ecosystems than systemic therapy. When medicines are administered systemically, they are usually excreted unchanged or as active metabolites in urine and faeces [78]. This leads to widespread soil and water pollution, especially in areas with intensive agricultural activity. Topical medicines, on the other hand, affect only the area where they are applied and are not absorbed into the body to the same extent, reducing the risk of spreading into the environment. For example, treating skin diseases or ectoparasites in a specific area can be just as effective with smaller amounts of active compounds and less exposure of the whole body [79]. Thus, eco-pharmaceutical management by reducing the amount of antimicrobials, antiparasitics, and anti-inflammatory drugs that are released into the environment could be supported. Also, avoiding systemic residues in manure or wastewater reduces the chances of antimicrobial resistance selection and harm to other species [80]. Topical medicines are in line with the sustainability goals of the One Health initiative because they promote animal health while keeping the environment safe, as long as they are used responsibly and monitored for residues.

## 3. Inflammation in Animals: Causes and Consequences

Understanding the pathophysiology and clinical spectrum of inflammatory conditions in companion and farm animals is a requirement for therapeutic decision-making and a fundamental element of the One Health framework. Inadequately managed inflammatory skin conditions drive increased and often systemic drug use, generate pharmacological residues that enter the food chain and accumulate in the environment, and compromise animal welfare in ways that increase susceptibility to secondary complications relevant to public health [81]. Consequently, the scientific justification for the selection of targeted, locally applied therapies that minimise these risks while maximising animal welfare is provided by a comprehensive comprehension of the underlying disease mechanisms.

Acute pain-related inflammatory responses arise from either sudden or continuous exposure to harmful stimuli and the interplay between pro-inflammatory and anti-inflammatory mediators. They function as a means of rehabilitation from an injury, trauma, sepsis, or infectious disease [82,83]. The improper management of acute inflammation can lead to the development of chronic conditions and persistent discomfort. The classifications of acute and chronic are primarily determined by a temporal framework, generally spanning from several days (acute) to weeks (chronic) [84]. Additionally, there could be variations in the severity of pain linked to these various time periods; chronic pain, for instance, might be less severe yet last longer.

Pain, a primary indicator of inflammation, is associated with elevated core and surface temperatures at the injury site, an aspect that can be assessed using infrared thermography, resulting from an association between pro-inflammatory mediators and nociceptors [85]. At the site of inflammation, various mediators, including prostaglandins, prostacyclin, interleukins (IL), leukotrienes, cytokines, histamine, serotonin, bradykinin, and tumour necrosis factor-alpha (TNF-α), facilitate vasodilation, enhance blood flow, and increase vascular permeability. These processes promote leukocyte emigration, chemotactic migration to the injury site, and the release of additional chemical mediators, resulting in clinical manifestations such as redness, swelling, pain, loss of function, and localised heat [86].

Dermatitis refers to a broad category encompassing various forms of skin inflammation. This term is commonly employed to refer to a skin condition prior to obtaining a definitive diagnosis [87]. Skin inflammation can arise from various factors, such as external irritants, burns, allergens, trauma, and infections, which may be bacterial, viral, parasitic, or fungal. The skin reacts to various triggers, which can present as a combination of symptoms, including itching, scaling, abnormal redness, thickening, and hair loss. The typical development of a skin condition includes an initial trigger that leads to the formation of boils, scabs, scales, or blisters [88]. Pruritus is a common symptom in various skin diseases and frequently arises due to secondary infections. Addressing dermatitis necessitates identifying the underlying cause and managing any secondary infections or complications that may arise. An analysis of the pet’s history, along with a thorough physical examination, can more accurately identify the issue. Dermatitis is most effectively managed using pharmacological agents administered topically, directly to the affected skin areas, as this route of administration allows high local drug concentrations at the site of inflammation while minimising systemic exposure and potential adverse effects. Topical administration is generally preferred for dermatitis because it enables targeted action, rapid symptom relief, and a reduced risk of systemic toxicity compared with oral or injectable formulations [89].

Acute-phase proteins (APPs) are serum proteins that are derived from the liver and undergo concentration fluctuations in response to inflammation, infection, or injury. The systemic response of the injured animal, which is marked by pyrexia, leucocytosis, endocrine changes, and the redistribution of trace elements, is mediated by pro-inflammatory cytokines (TNFα, IL-6, and IL-1). The changes in serum protein composition that occur after tissue damage are a component of this response [90,91]. Collectively, these responses have significance for the prevention of tissue injury and the improvement of the repair and resolution processes. The assessment of APPs provides a clinically applicable method for detecting inflammation.

Table 1 summarises the most common inflammatory conditions in dogs that can be treated with topical medications. These include problems with the skin, ears, anal tissue, legs, and breasts.

Most of the conditions shown in Table 1 are common skin conditions, such as atopic dermatitis, pyoderma, or *Malassezia dermatitis*. Their causes range from hypersensitivity reactions (type I and IV) to microbial overgrowth (bacterial, fungal, yeast) and immune-mediated pathologies, as well as trauma-related and idiopathic causes. Despite the fact that these conditions are distinct, they typically exhibit comparable clinical symptoms, including irritation, erythema, hair loss, fluid accumulation, and crusting.

Accordingly, a moderate haptoglobin (Hp) reactivity is initiated by inflammation in the dog. This APP is highly susceptible to the effects of corticosteroids in dogs, resulting in elevated Hp levels following corticosteroid treatment and during naturally occurring hyperadrenocorticism. This attribute serves to mitigate the utilisation of this application in the context of inflammation surveillance, as the interpretation of tests is impaired by steroid treatment [112]. Moreover, in several species, including dogs, C-reactive protein (CRP) serves as a significant APP, with serum concentrations capable of rising swiftly from 100 mg/L in response to various infectious diseases, such as babesiosis, leishmaniosis, leptospirosis, parvovirus infection, and *E. coli* endotoxemia [113]. Serum amyloid A, although less studied than CRP, also rises significantly during parvovirus, *B. bronchiseptica*, and leishmaniosis infections. Finally, α1-acid glycoprotein has been associated with chronic infections and neoplastic conditions, showing elevated levels in parvovirus infection, babesiosis, *E. canis* infection, lymphoma, as well as in both carcinoma and sarcoma. These underscore the utility of APPs in the clinical evaluation of disease severity, progression, and therapeutic response in dogs [114].

Table 2 summarises the most common inflammatory diseases in cats that can be treated with topical medications. These include allergic, infectious, immune-mediated, parasitic, and reactive conditions.

Feline atopic skin syndrome, flea allergy dermatitis, and eosinophilic granuloma complex are mediated by hypersensitivity reactions that cause Th2-biassed immune responses. These frequently manifest as itching, miliary dermatitis, and eosinophilic lesions [98]. *Malassezia* dermatitis, bacterial pyoderma, and dermatophytosis are infectious skin illnesses that arise from excessive microbial proliferation or exploitation of a cat’s compromised immune system. They usually manifest as red skin, oily skin, crusts, or hair loss [130]. *Pemphigus foliaceus*, the predominant autoimmune dermatosis in felines, characteristically manifests with pustular and crusted lesions. Autoantibodies targeting antigens associated with desmosomes or other intercellular connector proteins are implicated in the pathogenesis of *Pemphigus foliaceus*, as their impairment leads to acantholysis, characterised by intercellular separation [120]. Other conditions include feline acne, allergic contact dermatitis, and miliary dermatitis. These responses are typically based on patterns that arise due to heightened sensitivity of the body. At the same time, otitis externa, parasitic dermatitis (such as that caused by *Notoedres cati*), and injection site granulomas, which go beyond conventional dermatoses and support the need for personalised topical therapies.

Table 3 summarises inflammatory diseases in pigs, sheep, goats, and cattle. These diseases can be caused by multiple factors, such as infections, parasites, phototoxic lesions, and injuries.

Dermatophytosis in cattle arises when *Dermatophilus congolensis* infiltrates the skin, a process exacerbated by humid conditions that compromises the skin’s integrity, resulting in exudative crusts and pain [151]. Light-induced dermatitis, or photosensitisation, is characterised by an increased sensitivity of the skin to sunlight. This is typically due to the presence of photodynamic agents or chromophores in the skin. These chromophores absorb light energy, which is converted into a high-energy state that inflicts injury on nearby proteins, nucleic acids, and membranes. This energy has the potential to directly damage molecules or produce reactive molecules that initiate or continue chemical processes in dermal components. The buildup of reactive oxygen intermediates and free radicals is likely the most significant contributor to dermal injury. All of this leads to necrosis, degeneration, and damage to the epithelial cells [152]. Papillomatosis manifests as inflamed, hyperplastic warts caused by bovine papillomavirus [153], while digital dermatitis is a bacterial disease that typically results in painful lesions on the soles of the posterior feet and is a significant cause of disability [135].

Taken together, the inflammatory conditions described across Table 1, Table 2 and Table 3 illustrate the clinical complexity of dermatological disease in both companion and food-producing animals. Critically, these conditions share a common One Health dimension: when left inadequately treated, they predispose animals to secondary infections with bacteria of zoonotic relevance, drive the use of systemic antimicrobials and thereby contribute to resistance selection, and in farm animal settings generate drug residues that can enter the food chain and persist in the environment. The choice of anti-inflammatory treatment strategy, its route of administration, pharmacokinetic profile, and residue risk, therefore, carries consequences that extend beyond the individual animal and into the domains of public health, food safety, and ecosystem integrity.

## 4. Systemic vs. Topical Anti-Inflammatory Treatments—Advantages and Disadvantages

The selection of an anti-inflammatory treatment strategy in veterinary medicine has direct implications for drug residues, environmental contamination, antimicrobial resistance, and human health, all of which are core concerns of the One Health framework. Evaluating systemic and topical approaches comparatively, therefore, requires consideration not only of therapeutic efficacy and safety in the target animal, but also of their broader impact across the human–animal-environment interface.

Systemic therapy is recommended for diffuse inflammation, diseases with an immune component, or locally inaccessible lesions. The advantage lies in its wide distribution and rapid action [154,155]. However, these same characteristics accentuate other risks: the substances can also affect healthy tissues, increasing the potential for adverse reactions, especially with prolonged administration [156]. In addition, the dosage must be optimised for each species, as the mode of metabolism varies considerably, especially between monogastric (companion) animals and ruminants. Similarly, one of the biggest problems with systemic treatment is its lack of tissue specificity. Systemic drugs act on many organs and systems, regardless of where the inflammation is located. They enter the circulation and spread throughout the body, thereby increasing the risk of side effects, especially when the drugs are used for a long time or in high doses [157]. Long-term use of corticosteroids can cause iatrogenic hyperadrenocorticism (Cushing’s syndrome), immune suppression, slow wound healing, muscle atrophy, liver disease, and polyuria/polydipsia [158]. Additionally, abruptly stopping corticosteroids after long-term use can cause hypoadrenocorticism, a life-threatening condition that stops the hypothalamic–pituitary–adrenal axis from working [159]. NSAIDs do not possess any immune side effects, but they do have well-known negative effects on the stomach, kidneys, and liver. Non-selective inhibition of COX-1-derived prostaglandins, which protect the stomach lining, can cause stomach ulcers and bleeding. This risk is even greater in animals that already have health problems or when NSAIDs are used in combination with corticosteroids [155,160]. NSAIDs reduce prostaglandin-mediated vasodilation in the kidneys, which can cause acute kidney damage, especially in dehydrated animals or those with other kidney problems. Hepatotoxicity has also been observed, but less frequently. This may be idiosyncratic or dose-dependent, depending on the drug and species [161,162].

For animals, the exposure to NSAIDs is reasoned to result in a range of undesirable consequences, for example, reproductive disruption, genotoxicity, oxidative stress, and teratogenic effects [163].

Furthermore, from a practical standpoint, systemic drug administration can be difficult in uncooperative or agitated animals, which may lead to discontinuation of therapy. These constraints highlight the need to review current treatment options and develop new topical therapies that reduce systemic exposure while being effective and beneficial to animals [164]. Another practical problem is compliance with clinical treatment. For some animals or species, repeated administration of drugs orally or by injection is difficult because of how they work, how they react to stress, or because of the animal’s anatomy [165]. Moreover, systemic anti-inflammatory medication can mask clinical signs without addressing the underlying cause, especially when the cause is an infection. This could make it take longer to obtain a clear diagnosis and the appropriate therapy for the cause.

In addition, systemic drugs raise serious concerns about drug residues in edible tissues of food-producing animals, which is why well-defined withdrawal periods and strict regulatory control are necessary [111,112,166]. For instance, several traditional NSAIDs are approved for use in animals raised for food. Due to the extensive usage of these medications, consumers may be at risk if remnants from the pharmaceuticals find their way into food [167,168]. In this direction, the occurrence of NSAID residues in milk and muscle samples was assessed using the developed method within the Croatian National Residue Control Plan, revealing quantifiable residues for diclofenac, ketoprofen, and salicylic acid, mostly in milk samples. The most abundant NSAID in the analysed samples was salicylic acid, which may enter the food chain and be present in various matrices due to its natural occurrence in plants as a phytohormone [169].

The idea of managing pain in veterinary medicine is gradually gaining popularity, prompting interest in developing novel methods. However, the range of medications available to veterinary pharmacologists remains rather limited. It is of the utmost significance to test novel human drugs and therapies in veterinary species [170,171]. Furthermore, in farm animals, veterinarians are commonly faced with the need to administer drugs to a species in a manner that is not in accordance with the label directions. When using pharmaceuticals in an extralabel manner, the veterinarian assumes legal responsibility for ensuring that the product is safe and efficacious and will not leave harmful residues in animal products intended for human consumption. From a One Health perspective, the widespread extralabel use of anti-inflammatory drugs in food-producing animals represents a systemic gap that extends beyond individual veterinary responsibility. When drugs are administered outside their approved indications, the absence of species-specific pharmacokinetic data and validated withdrawal periods makes it impossible to reliably predict residue depletion in edible tissues, milk, or eggs, creating blind spots in food safety monitoring systems that are designed to protect human health [166]. Furthermore, the lack of standardised data on extralabel use hampers regulatory authorities, public health agencies, and environmental scientists’ ability to conduct meaningful cross-sectoral risk assessments, which are a cornerstone of the One Health approach. Addressing this gap requires not only stronger pharmacovigilance frameworks but also active collaboration between veterinarians, regulatory bodies, and food safety authorities to generate the evidence needed to support safe, evidence-based therapeutic decisions across the entire food production chain.

However, there is still an insufficient amount of information about their pharmacokinetics in animals; however, it has been proven that acidic drugs (pKa 4–5), such as diclofenac, ibuprofen, ketoprofen, and more, seem to accumulate and persist in inflamed tissue, such as the synovial fluid of an inflamed joint. Because the administration of NSAIDs for medical and veterinary purposes is so vast, they are also regarded as environmental contaminants. Recent findings show NSAIDs residues can cause deadly threats for many animal species, such as Indian vultures, which die due to diclofenac poisoning [172].

This makes it difficult to organise treatments and means that thorough checks on the safety of medicines are necessary to ensure that food safety rules are not violated. However, it is important to note that the withdrawal periods required to eliminate the risk of residues of active substances in food are not known for all medicines administered to farm animals [173,174]. There are also several unknown variables in this area, such as in polytherapy with multiple active substances, where there is a risk of drug interactions that potentiate pharmacological effects.

For this reason, residues must be controlled, and appropriate analytical methods must be developed to ensure compliance with EU legislation. To protect consumer health, the European Union establishes MRLs for pharmacologically active substances used in food-producing animals where necessary, based on scientific risk assessment. The numerical values of MRLs vary widely depending on the substance, target species, and edible tissue, and may range from very low microgram-per-kilogram levels to higher concentrations, or may not be required at all for certain substances [175].

The monitoring of residues in food of animal origin is an important task for both human and animal health. Although the risk associated with the intake of drugs with food is low, it must still be ensured that the levels of these substances will not exceed the limits introduced in regulatory documents. Despite some NSAIDs being registered as veterinary drugs, only a few have defined MRLs and are allowed for use in food-producing animals [176]. The EFSA reports that out of 24,387 samples (milk, muscle) analysed by European laboratories participating in the official food control in 2019, 46 (0.19%) were found to be non-compliant [177].

Additionally, the inappropriate use of systemic medications, particularly antibiotics, can promote antimicrobial resistance. Concomitant use of anti-inflammatory drugs may modify infection dynamics or antibiotic efficacy, potentially influencing resistance selection in some contexts. Some NSAIDs have the potential to encourage the emergence of cross-resistance. This entails choosing resistance mechanisms, multidrug efflux pumps being a notable example, that also give resistance to antibiotics. Through a process known as transformation, bacteria can also acquire new resistance mechanisms by taking up a plasmid that expresses resistance factors. Another drug that can boost transformation efficiency and hence raise acquired resistance is diclofenac [178]. In general terms, a minor drug contamination could not constitute a public health hazard. However, the widespread use of medications may increase the risk of adverse effects from residues on consumers, such as the development of antibiotic resistance and allergic reactions. Because of this, it is important to use caution while administering medicines in order to avoid contaminating feed [179].

From an environmental perspective, systemic drugs might contribute significantly to ecosystem pollution. Active metabolites excreted in urine or faeces can accumulate in soil and wastewater, persisting for long periods and affecting non-target species. This problem is particularly relevant in industrial farms, where repeated treatments can lead to ecotoxicological outbreaks [180]. A telling example is diclofenac, which has been linked to declines in populations of scavenging vultures. These birds fed on treated carcasses and were exposed to toxic risk [181]. In the same direction, aquatic ecosystems, particularly river waters, are critically important for ecological processes and provide a variety of significant benefits for human well-being [182], as they serve as reservoirs of antimicrobial resistance. They are believed to constitute a significant pathway for the dissemination of resistant components, nevertheless. Antibiotic residues have been discovered in natural river water worldwide, and their concentrations are rapidly increasing. This is largely due to the widespread use of antibiotics in human and veterinary medicine. Antibiotics found in faeces can cause antibiotic pollution and antibiotic-resistant bacteria [183]. Accordingly, Skocovska et al. have shown the numerous occurrences of sulfamethoxazole, ibuprofen, naproxen, diclofenac, and paracetamol (acetaminophen). A statistically significant negative correlation between river flow rate at the monitored locations and residue concentration was found for ibuprofen, naproxen, diclofenac, and paracetamol. The most significant findings of the monitored drug residues were observed in samples from small streams below larger urban settlements with a hospital or other health facilities [184]. Furthermore, high levels of common NSAIDs, including aspirin, diclofenac, naproxen, ibuprofen, and ketoprofen, were found in wastewater up to 2,747,000 ng L^−1^. Gene toxicity, endocrine disruption, motor problems, organ destruction, bodily deformations, and photosynthetic corruption are all possible outcomes of NSAIDs in water. Animals, vegetation, and human health are all negatively impacted when NSAIDs are released into the environment. In terms of human health, consuming NSAIDs may raise the risk of renal damage, which might lead to harmful liver issues and raise the risk of stroke and cardiovascular mortality. NSAID chemicals can prevent the growth and development of green algae, *Vigna unguiculata*, and *Oryza sativa* L. The amount of photosynthetic pigments and root and cell damage is often affected by how NSAIDs are metabolised in plants [163].

On the other hand, topical anti-inflammatory drugs in veterinary medicine are effective because they achieve high local concentrations with minimal systemic absorption. This significantly reduces the risk of general toxicity, including adrenal toxicity (in the case of corticosteroids) or gastric toxicity (in the case of NSAIDs) [185,186]. In addition, topical treatment is preferable for animals that cannot receive oral or injectable medications, which increases therapeutic adherence on the part of owners and contributes to animal welfare [16]. On farms or in food-producing animals, topical administration may reduce the risk of drug residues, especially if applied to intact skin and in accordance with approved withdrawal periods [187].

However, there are limitations: topical efficacy is lower in deep or systemic inflammatory conditions and depends on the integrity of the skin barrier, the characteristics of the formulation (lipophilicity, vehicle), and the behaviour of the animal (rubbing, grooming) [16]. In large animals or in intensive systems, repeated application can be laborious. In addition, inappropriate use of topical formulations, especially antimicrobials, may lead to the development of local resistance [188].

It is important to note that specialists in the field should consider systemic and topical therapies as complementary strategies. In severe or generalised inflammation, systemic treatment cannot be avoided, but combination with topical agents can optimise the therapeutic effect, reduce the dose of medication administered, and minimise the impact on the environment.

From a One Health perspective, topical anti-inflammatory therapy, when rationally selected, appropriately formulated, and monitored for residues, represents the route of administration most closely aligned with the principles of therapeutic precision, environmental stewardship, and cross-sectoral responsibility. The development of innovative formulation strategies is a pertinent strategy to the broader imperative of delivering effective veterinary care while safeguarding public health and the environment.

## 5. Recent Advancements in the Development of Topical Veterinary Formulations

### 5.1. Active Pharmaceutical Ingredients

Currently, the development of topical dosage forms for veterinary medicine involves a wide range of active substances, broadly grouped in two categories: chemically synthesised molecules, structurally modified to enhance local potency and limit systemic absorption, and bioactive compounds of natural origin, extracted from plants or other biological sources, which could be sustainable and low environmental impact solutions [189,190,191,192]. Corticosteroids, NSAIDs, and immunomodulators remain first-line treatments, but their pharmacokinetic constraints and risks related to adverse reactions or drug residues in the food chain have stimulated interest in alternatives with a more favourable toxicological profile [193,194,195].

The diversification of active pharmaceutical strategies in topical veterinary therapy reflects a transition from conventional broad immunosuppression toward targeted, locally confined, and sustainability-oriented interventions. Beyond classical corticosteroids and NSAIDs, recent developments include molecularly optimised prodrugs, selective cytokine modulators, pro-resolving lipid mediators, antimicrobial peptides, barrier-replenishing lipids, and bioactive compounds of natural origin. These approaches aim to enhance cutaneous selectivity, reduce systemic exposure and residue risk, and, where possible, align pharmaceutical innovation with antimicrobial stewardship and environmental responsibility. The main emerging strategies, their molecular targets, developmental rationale, and clinical implications are summarised in Table 4.

With regard to topical corticosteroids, recent research has focused on the development of derivatives with optimised pharmacokinetic profiles, such as hydrocortisone aceponate. This lipophilic ester has an increased skin penetration and is locally activated by tissue esterases, while systemic absorption remains limited, reducing the risk of suppression of the hypothalamic–pituitary–adrenal axis [215]. The use of prodrugs or more lipophilic esters provides a balance between the potency of the anti-inflammatory effect and the risk of cutaneous atrophy, an effect commonly seen with classical glucocorticoids. The development of topically administered NSAIDs has as its main objectives selectivity for COX-2 and reduction in systemic exposure to limit the known risks of gastrointestinal ulceration and nephrotoxicity associated with oral administration. Modern formulations utilise ionic pro-carriers or ion pairing systems, which improve transdermal permeability without requiring high concentrations of irritant organic co-solvents [216]. Other strategies, such as controlled supersaturation or the use of nanocrystals, can improve cutaneous flux and achieve local therapeutic concentrations in inflamed tissues with minimal plasma exposure [217]. These advantages are especially relevant in dogs and horses, where chronic musculoskeletal pathologies are frequently treated with NSAIDs. In addition, differences in skin thickness and vascularisation between species necessitate specific ex vivo tissue testing to validate the penetration profile and residue risk.

Immunomodulators represent an emerging direction with off-label uses already reported in veterinary practice [218]. Tacrolimus, a calcineurin inhibitor, has been adapted from human dermatology and successfully applied in dogs with refractory atopic dermatitis and in cats with eosinophilic granuloma-like lesions [219]. Transfer of knowledge from human medicine is useful, but interspecies differences require further evaluation since skin permeability is different from that in humans, and for some species, especially cats, the metabolic profile is restrictive [220].

A recent direction in veterinary dermatopharmacology is the use of specialised mediators of inflammation resolution (lipoxins, resolvins, protectins) derived from polyunsaturated fatty acids. In contrast to corticosteroids or NSAIDs, these compounds do not directly inhibit classical inflammatory cascades (COX, LOX), but accelerate endogenous resolution processes by stimulating phagocytosis of cellular debris, reducing neutrophilic infiltrate and restoring tissue homeostasis. Technologically, their low stability requires formulation in protective vehicles, such as liposomes, nanostructured lipid carriers or thermogelling poloxamers, to prevent oxidation and degradation [221]. The advantage is that these molecules do not induce immunosuppression and do not favour secondary infections.

An emerging area in veterinary topical therapy is the use of adjuvants aimed at replenishing the skin barrier, in particular the intercellular lipid complex, in order to reduce the need for anti-inflammatory or antimicrobial drugs [222]. Another increasingly important area of research is the use of bioactive compounds of natural origin, extracted from medicinal plants, algae, fungi, or by-products of the agri-food industry [223]. Compared to synthesised molecules, these compounds generally exhibit superior biodegradability and a more favourable toxicological profile, which gives them an advantage in the context of One Health [19]. Importantly, the use of plant sources to obtain bioactive compounds is not limited to the therapeutic value of the molecules, but can bring additional benefits when sustainable processing methods are applied [224,225]. For example, the extraction of polyphenols from agri-food by-products, such as grape skins, tomato skins, or citrus peels, by green technologies such as ultrasound-assisted extraction, supercritical fluid extraction, and enzymatic methods, allows the recovery of active principles with anti-inflammatory and antioxidant effects, while reducing the environmental impact of organic waste [226]. Thus, the need for the use of toxic organic solvents can be decreased, and the risk of contamination of both the final product and the ecosystem can be reduced. In this direction, the use of these methods offers a number of advantages: they increase the ethical acceptability of veterinary pharmaceuticals, reduce the environmental footprint of the manufacturing process, and offer alternatives to new active substances with lower toxicological effects. The integration of circular economy principles through the valorisation of agricultural and agro-industrial waste could offer new ways to develop formulations that not only support animal health and reduce human exposure to residues, but also contribute to the conservation of natural resources [227,228].

However, the use of natural bioactive compounds in veterinary topical therapy requires a prudent and scientifically based approach. At present, there is insufficient evidence that a plant extract can match the potency of established synthetic molecules in controlling severe inflammation [105,229]. However, these substances may be of value in the early stages of inflammation, in mild dermatitis, superficial lesions, or as adjuvants, when the aim is to restore physiological balance rather than to suppress the immune response intensively. Their use in such contexts limits exposure to synthetic molecules with systemic potential and improves safety, particularly in sensitive, elderly, or chronically ill animals.

Additionally, despite the mechanistic plausibility and promising in vitro and preclinical data supporting the anti-inflammatory activity of polyphenols, flavonoids, and algal polysaccharides, the transition to clinically validated, species-specific applications remains limited. A comprehensive literature review by van Amersfort et al. (2023), covering 64 peer-reviewed publications on nutraceuticals in canine dermatological immune-mediated inflammatory diseases, concluded that only minor evidence exists for several compounds, including essential fatty acids, niacinamide, and probiotics, while major clinical evidence for most natural bioactive substances is currently lacking [230]. For example, in the specific context of topical application, compounds such as laminarin, a β-glucan polysaccharide from brown marine algae, have demonstrated anti-inflammatory activity in oxazolone-induced atopic dermatitis-like mouse models, significantly reducing mast cell infiltration, epidermal thickening, and serum IgE levels; however, to date, these findings do not appear to have been translated into controlled veterinary clinical trials [212]. Likewise, flavonoids such as naringin have shown efficacy in DNCB-induced murine atopic dermatitis through inhibition of the JAK-STAT signalling pathway. Although topical tacrolimus was used as a positive control in the study, direct equivalence to tacrolimus was not definitively established. In addition, companion-animal data specifically addressing topical dermatologic pharmacokinetics, safety, and controlled clinical efficacy remain lacking [231]. In a broader context, Bannach et al. (2025) reported that a novel antibiotic-free topical formulation combining antimicrobial peptides with encapsulated plant extracts was effective in the management of canine otitis externa, providing proof-of-concept for the use of plant-derived bioactives within multimodal veterinary topical therapy [105]. Nevertheless, these encouraging findings must be distinguished from established therapies. In contrast to natural bioactive compounds, corticosteroids such as hydrocortisone aceponate, NSAIDs, calcineurin inhibitors such as tacrolimus, and JAK inhibitors such as oclacitinib are supported by multiple randomised, controlled clinical trials in target veterinary species, with well-defined efficacy endpoints, dose–response data, and pharmacovigilance records. Natural bioactive compounds, therefore, currently represent a complementary and adjuvant strategy with a favourable safety and ecotoxicological profile, most applicable in mild-to-moderate inflammatory conditions, in maintenance therapy, or as drug-sparing adjuncts. Therefore, it is important to note that these approaches do not aim to replace established therapies but to optimise their safety profile, offering new perspectives for integrating sustainable formulations with low toxicity risk into veterinary practice in accordance with One Health principles. Moreover, research in this area should be encouraged to strengthen the scientific basis for using these formulations in current clinical practice.

### 5.2. Vehicles and Excipients

Vehicles and excipients are important pharmaceutical components in determining the clinical performance and safety of a dosage form [232]. In the context of developing new dosage forms for the topical treatment of animals, the ideal vehicle should combine pharmacologic efficacy with minimal ecotoxic impact [233].

A current trend is the use of systems without organic solvents or with low levels of volatile organic compounds, as these can cause skin irritation, occupational exposure, and air pollution [234]. Replacing ethanol, isopropanol, or toxic glycols with biodegradable natural excipients such as medium-chain triglycerides, plant-derived isopropyl myristate, lauric acid esters, or lecithin phospholipids could provide robust technological alternatives [235]. At the same time, reducing wash-off is necessary to limit contamination of the aquatic environment. Vehicles with film-forming properties or lamellar structures (organogels, inverse emulsions, lipid microemulsions) provide local retention and prolonged release of the active substance, decreasing transfer to water or soil [236,237].

In terms of degradability, excipients based on natural lipids, biopolymers, or short-chain aliphatic esters metabolise to non-toxic metabolites, preventing bioaccumulation [238,239]. At the same time, preservative substances should be designed to exert minimal pressure on the skin microbiome [240]. According to the European Medicines Agency (EMA) Note for Guidance: Development Pharmaceutics for Veterinary Medicinal Products (EMEA/CVMP/315/98), the selection and use of excipients in veterinary medicinal products must be scientifically justified within the pharmaceutical development section of the marketing authorisation dossier. Each excipient included in a formulation should have a clearly defined technological function and be compatible with both the active substance and other formulation components, supported by appropriate experimental data. The quality and quantity of excipients must be justified in relation to their role in the formulation and the manufacturing process, while their physicochemical characteristics and potential interactions should be evaluated during development. When novel excipients or unconventional formulation constituents are introduced, such as new matrices, permeability enhancers, or propellants, comprehensive information on their composition, function, and safety must be provided, and such substances may be assessed similarly to new active ingredients unless prior regulatory acceptance exists for comparable uses. In addition, it is emphasised that excipient selection must consider stability, compatibility, and safety throughout the product’s shelf life, as well as the intended route of administration and dosage form, ensuring that the final formulation consistently meets the required quality and performance specifications [241].

In parallel with the optimisation of conventional vehicles, there is growing interest in biodegradable and renewable polymeric materials produced by sustainable processes. These have the potential to replace synthetic excipients that are persistent in the environment and to valorise biomass by-product streams, reducing the environmental footprint of pharmaceutical production [242,243]. A representative example is chitosan, a natural polymer derived by deacetylation of chitin, which can be obtained from marine waste, shrimp exoskeletons, crab exoskeletons, or *Rapana venosa*, generated by the seafood industry [244]. Chitosan is a cationic polymer with film-forming, bioadhesive, and antimicrobial properties, attributed to its free amino groups, which interact electrostatically with bacterial phospholipids. Numerous studies have confirmed its biocompatibility and lack of systemic toxicity at relevant topical doses, and enzymatic degradation by endogenous chitinases generates metabolites (glucosamine, N-acetylglucosamine) without toxicity [245,246,247,248]. Chitosan can be integrated in mucoadhesive hydrogels (by solubilization in weak organic acids such as lactic or acetic acid), cationic nanoparticles for controlled release, bioadhesive films, microspheres, or membranes for wound healing [249,250,251]. In otic and dermatological formulations, chitosan exhibits several functions as a viscosity agent, suspension stabiliser, and penetration promoter, by its ability to transiently modulate tight junctions in the stratum corneum without causing irritation [252]. Intrinsic antimicrobial properties allow for the reduction in the concentrations of synthetic preservatives, thus decreasing the impact on the skin microbiome and the risk of selection for bacterial resistance.

Alginate is an anionic polysaccharide extracted from the cell walls of brown algae (*Laminaria*, *Macrocystis*). It is completely biodegradable and non-toxic, exhibiting gelling properties in the presence of divalent ions. It is used for the production of controlled-release hydrogels, bandages, and microcapsules [253,254]. The use of alginate valorises renewable marine biomass and allows the replacement of synthetic excipients, reducing chemical pollution. Furthermore, the polymeric structure favours local retention of active ingredients and protection of skin lesions without the need for harsh preservatives, which contributes to reducing the environmental impact and the risk of antimicrobial selection [255,256].

Pectin is a heteropolysaccharide obtained mainly from the peels and waste of apple or citrus fruits. It is biodegradable, biocompatible, and forms transparent gels or films in the presence of Ca^2+^ ions, which makes it attractive for mucoadhesive preparations and controlled-release systems [257,258]. The use of pectin valorises agro-industrial streams that would otherwise be treated as organic waste, and its moisturising and skin-barrier-protective properties could reduce the need for synthetic excipients and facilitate the development of sustainable pharmaceutical forms by reducing the risk of producing ecotoxic residues in the food chain [259,260].

Pullulan is a polysaccharide produced by fermentation of the fungus *Aureobasidium pullulans*. It is biodegradable, transparent, and forms tough, bioadhesive films. It is used in oral films, film-forming solutions, and microcapsules, is non-toxic, and is approved for food use. Fully biodegradable, pullulanic films can be used as vehicles for topical active substances or as protective coatings for wounds with minimal environmental impact. Another example is polylactic acid (PLA), a biodegradable polyester obtained from the fermentation of starch, followed by polymerisation. It is biocompatible and degrades to lactic acid, a physiological metabolite. PLA can be used for nanoparticles, microspheres, and controlled-release films [261,262].

On the other hand, the selection of excipients for veterinary topical formulations requires special attention, as interspecies differences can transform a safe vehicle in humans into a potentially toxic compound for animals [263]. Propylene glycol, used as a solvent and humectant, is metabolised to a limited extent in cats due to hepatic glucuronyltransferase deficiency, resulting in accumulation and risk of oxidative hemolysis, Heinz body formation, and central depression [264]. Similarly, terpenes (menthol, camphor, eucalyptol), used as penetration promoters, can induce neurotoxic reactions in cats through rapid absorption and poor hepatic metabolism [265]; they can be replaced by milder promoters such as fatty acids or natural phospholipids. In food-producing species, excipient selection requires particular attention because formulation components capable of generating residues in edible tissues must comply with the European regulatory framework established by Regulation EC No 470/2009, which mandates the scientific evaluation of pharmacologically active substances and the establishment of MRLs to ensure the safety of food of animal origin [59]. In this context, the transition to biodegradable, non-toxic, and volatile excipients is no longer just a technological option, but also an ethical and regulatory requirement in the spirit of One Health.

### 5.3. Pharmaceutical Formulations and Drug Delivery Systems

Currently available veterinary topical pharmaceutical formulations are mainly sprays, lotions, creams, and ointments, with the addition of otic gels and foams to a lesser extent. These products are generally formulated with corticosteroids, NSAIDs, antifungals, or antibiotics and are designed for easy application and even coverage. However, they have important limitations, such as excipient volatility, the need for frequent applications, and the difficulty of adherence to hair-covered integuments or wet surfaces. Medicated collars and spot-on solutions are pharmaceutical forms used for the administration of pesticides, but most current products have an unintended systemic profile, due to dermal absorption and distribution through sebum, causing the active substance to be excreted into the aquatic environment by bathing, washing, or contact with soil, and contributing to the contamination of ecosystems [266,267]. Efforts are needed to reconfigure these formulations by achieving a predominantly topical rather than systemic action, by modifying lipid vehicles and the rate of diffusion through the epidermis. Consideration must also be given to the possibility of reducing human exposure and contamination of the aquatic environment, a significant problem associated with the use of conventional antiparasitics.

For these reasons, current research is focusing on advanced formulations tailored to animal behaviour and the anatomical particularities of the species to optimise therapeutic efficacy and reduce environmental and occupational impacts (Table 5).

Topical films and in situ-forming film systems are developed for hard-to-reach areas (interdigital, perianal, skin folds) [288,289]. They combine biodegradable polymers such as PLA, pullulan, and PVA with natural plasticisers, forming a protective barrier that gradually releases the active substance. This reduces the need for preservatives, and pharmaceutical formulations can be obtained with complete degradability and limited washout losses.

Transdermal or occlusive patches are especially tested for musculoskeletal treatments in dogs [290]. Solid lipid multilayer systems allow constant release and reduce systemic exposure [16]. However, their use has to be justified, as non-biodegradable polymeric carriers (polyurethane, silicone) generate persistent waste, and the risk of accidental ingestion by other animals is not negligible.

Despite their pharmacotechnical advantages, the advanced drug delivery systems discussed in this section present significant economic challenges that must be acknowledged in the context of routine veterinary practice. The production of nanostructured lipid carriers, poly-lactic-co-glycolic acid nanoparticles, liposomes, and in situ gelling systems involves complex multi-step manufacturing processes, specialised equipment, stringent quality control requirements, and, in the case of lipid-based nanocarriers, particular demands regarding stability, sterilisation, and cold-chain storage. All of these substantially increase production costs compared to conventional topical formulations such as creams, ointments, and sprays [291,292]. These cost barriers represent an important obstacle to the routine implementation of advanced delivery systems in veterinary medicine, particularly in large-animal practice and resource-limited settings, where the economic value per treatment unit is subject to strict market constraints [58]. Furthermore, scaling up from laboratory-scale synthesis to industrial manufacturing introduces additional technical challenges, including reproducibility of particle size distribution, batch-to-batch consistency, and avoidance of aggregation, that further inflate development costs and extend time-to-market timelines [293]. In companion animal practice, where clients may accept higher costs for advanced therapeutics, the economic barrier is relatively lower; however, in food-producing animal medicine, cost-effectiveness is a primary determinant of adoption, and the widespread use of nanoformulated topical products for conditions such as mastitis or digital dermatitis remains economically impractical in its current form [294]. Addressing these challenges will require the development of scalable, solvent-free, and continuous manufacturing processes, as well as the strategic use of sustainably sourced, bio-derived excipients, which offer not only a more favourable environmental profile but also potential cost reductions through valorisation of agro-industrial by-products. Long-term, the integration of One Health-aligned economic models into veterinary pharmaceutical development, accounting for the full cost of systemic drug residues, antimicrobial resistance, and environmental remediation, may help reframe advanced topical delivery systems not as a premium cost, but as a cost-effective investment in ecosystem and public health protection [58].

## 6. Regulatory Challenges for Topical Anti-Inflammatory Veterinary Products

**Figure 2:**
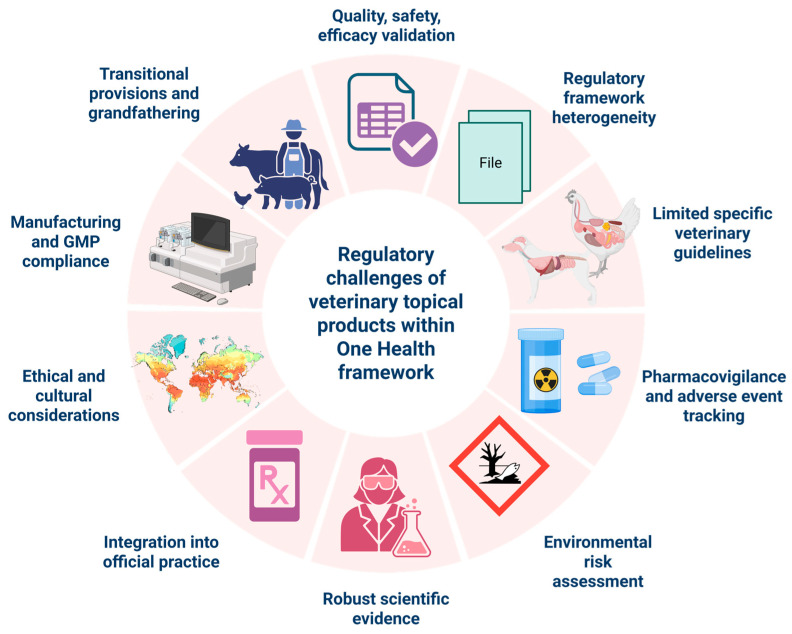
Regulatory challenges associated with the development and implementation of veterinary topical medicinal products within the One Health framework (Created with Biorender.com).

The need to simultaneously address animal health, public health and environmental protection within the One Health paradigm is underscored by the complicated regulatory environment controlling veterinary pharmaceuticals. As shown in Figure 2, several regulatory issues beyond conventional pharmaceutical evaluation can impact the development and use of veterinary topical treatments. These challenges include the need for sound scientific data to support both clinical efficacy and environmental safety; adherence to manufacturing standards and good manufacturing practices; and stringent validation of quality, safety, and efficacy. The institutions involved in regulation at this level also need to consider pharmacovigilance mechanisms for tracking adverse events, environmental risk assessment and incorporation of new products into standard veterinary practice. The lack of veterinary-specific guidelines for advanced topical formulations, the diversity of international regulatory regimes and transitional measures impacting older medicinal products contribute to the complexity of the regulatory environment. Regional variations in regulatory application and adoption may also be influenced by ethical, cultural and socioeconomic issues. Legislative fragmentation and the absence of consistent criteria across jurisdictions have posed challenges for the regulation of topical anti-inflammatory veterinary medicinal products.

Regulation EU 2019/6 on veterinary medicinal products sets the general framework in the European Union, replacing Directive 2001/82/EC [295]. It necessitates an evaluation of environmental risk, efficacy, safety, and quality. Nevertheless, it is important to note that the Regulation does not establish a specific simplified regulatory category for products with minimal systemic absorption. Rather, data requirements must be scientifically justified on a case-by-case basis. The demonstration of compliance with established MRLs may be complicated by the variability in transcutaneous absorption and limited residual depletion data in food-producing animals, which can make it difficult to establish withdrawal periods for topical products.

The establishment of MRLs is another regulatory challenge. A harmonised EU-level framework for the scientific risk assessment and classification of pharmacologically active substances used in food-producing animals is established by Regulation EC No 470/2009 [59]. Nevertheless, the determination of withdrawal periods and marketing authorisation decisions may be complicated by limited or difficult-to-interpret residual depletion data for certain topically administered substances, resulting from variability in dermal absorption. Although the FDA Centre for Veterinary Medicine in the United States implements comparable principles for residue evaluation and target animal safety (GFI #3 and GFI #185) [296,297], variations in regulatory interpretation, tolerance listings, and data requirements may result in certain products that have been authorised in the EU not being approved for the US market.

Quality by Design principles, as outlined in ICH Q8, Q9, and Q10, are recommended but not mandatory in the context of quality and manufacturing [298]. Conventional development methodologies are still considered permissible. Rather than prescribing specific development models, these guidelines prioritise scientific comprehension, risk management, and lifecycle control. Veterinary medicinal products, including complex topical systems, are subject to general pharmaceutical quality requirements. However, there is a dearth of dosage-form-specific guidance for advanced veterinary formulations, such as nanoemulsions or in situ film-forming systems. As a result, these products frequently necessitate more rigorous scientific justification, and regulatory expectations may differ based on the procedure and authority.

Another deficiency is the absence of harmonised, dosage-form/indication-specific guidance on the validation of clinical endpoints in target species. However, Regulation EU 2019/6 and Annex II establish general requirements for veterinary clinical trials, including controlled designs, statistical principles, and conduct in accordance with established VICH good clinical practice [295]. However, they do not standardise disease-specific clinical scoring instruments, which can complicate cross-study comparability and comparative efficacy assessments.

A new concern is the ambiguous classification of veterinary medicinal products, biocidal products, feed additives, and other product categories. Regulation EU 2019/6 explicitly acknowledges these boundary issues and establishes mechanisms for case-by-case determinations (Coordination Group recommendations and Commission decisions). Pharmacovigilance capture and Union-level traceability mechanisms may be compromised when products with purported physiological effects are not included in the veterinary medicinal product framework. This is especially pertinent from a One Health perspective, as the Regulation explicitly incorporates environmental and resistance risks into the benefit–risk assessment.

Several regulatory and environmental challenges may be addressed through the development of new anti-inflammatory therapeutics for veterinary use in accordance with One Health principles. The predictability of environmental and toxicological outcomes can be enhanced through the integration of eco-pharmacology, green chemistry strategies, and early-stage integrated risk assessment. This, in turn, facilitates compliance with the environmental risk assessment and benefit–risk framework established under Regulation 2019/6.

The utilisation of biodegradable carriers and excipients with well-characterised toxicological profiles has the potential to reduce uncertainty in residue and environmental exposure assessments and facilitate a more efficient evaluation of depletion and ecotoxicity data. However, regulatory requirements are still substance- and context-specific. Similarly, controlled-release topical platforms (e.g., solid lipid nanoparticles, nanostructured lipid carriers, mucoadhesive gels, biodegradable films) that are intended to reduce systemic exposure may aid in the scientific justification of withdrawal periods in food-producing animals under Regulation EC No 470/2009, provided that residue depletion data verify compliance with established MRLs.

The robustness of manufacturing and control strategies can be further enhanced through the implementation of lifecycle risk management and Quality by Design principles. Furthermore, the utilisation of sustainable excipients and bio-derived materials may enhance environmental profiles that are in accordance with environmental risk assessment requirements and public health objectives, while solvent-free or low-volatile organic compounds manufacturing processes may mitigate occupational and environmental exposure risks and facilitate compliance with broader EU chemical safety frameworks.

## 7. Conclusions

Topical anti-inflammatory therapies represent an important component of veterinary pharmacotherapy, particularly for dermatological and localised inflammatory conditions in companion and food-producing animals. As discussed in this review, their rational use can reduce systemic drug exposure, limit adverse effects, and contribute to improved therapeutic precision. Within the One Health framework, the development of these products requires careful consideration of multiple interconnected factors, including pharmacological efficacy, animal welfare, environmental safety, and public health protection. Particular attention must be given to formulation design, excipient selection, environmental risk assessment, residue control in food-producing species, and post-marketing pharmacovigilance. At the same time, regulatory heterogeneity, limited veterinary-specific guidance for advanced topical systems, and gaps in clinical evidence remain important challenges. Future progress will depend on multidisciplinary collaboration and the integration of pharmaceutical innovation with harmonised regulatory and environmental safety strategies to ensure the responsible use of topical veterinary medicines.

## Figures and Tables

**Table 1:** Common inflammatory conditions in dogs.

Disease	Pathophysiological Mechanism	Clinical Outcomes	References
Canine atopic dermatitis	IgE-mediated hypersensitivity to environmental allergens; Th2-skewed immune response; impaired skin barrier	Pruritus, erythema, lichenification, secondary infections, alopecia, chronic relapsing dermatitis	[89]
Flea allergy dermatitis	Type I and IV hypersensitivity to antigens in flea saliva	Intense pruritus, papules, crusts, alopecia, distribution over the lumbosacral area, caudal thighs, and abdomen	[92]
Contact dermatitis (allergic/irritant)	Delayed-type (Type IV) hypersensitivity in allergic form; direct cytotoxicity in irritant contact dermatitis	Erythema, vesicles, pruritus or pain, alopecia, often on hairless or poorly haired skin (e.g., abdomen, paws)	[92,93]
Canine pyoderma (superficial)	Bacterial infection (commonly *Staphylococcus pseudintermedius*) with inflammation confined to the epidermis	Papules, pustules, epidermal collarettes, scaling, pruritus, alopecia	[90,91,94]
Seborrheic dermatitis	Dysregulation of keratinisation; possibly secondary to endocrine or dietary imbalances	Greasy or dry scaling, erythema, odour, pruritus	[95]
Autoimmune dermatoses (e.g., pemphigus foliaceus)	Autoantibody-mediated attack on epidermal desmosomal proteins (e.g., desmoglein-1)	Crusting, pustules, erosions mainly on face, ears, and footpads; often symmetrical	[96]
Hot spots (acute moist dermatitis)	Self-inflicted trauma due to an underlying pruritic or painful condition; bacterial overgrowth	Localised erythematous, exudative, painful lesions with alopecia and moist crusting	[97]
*Malassezia* dermatitis	Overgrowth of *Malassezia pachydermatis* in predisposed dogs, often secondary to allergic disease	Pruritus, erythema, greasy skin, hyperpigmentation, lichenification	[98,99,100]
Canine lupus erythematosus (discoid)	Immune complex deposition and cytotoxic T-cell-mediated keratinocyte apoptosis	Depigmentation, crusts, erosions on the nasal planum, periocular, and pinnae areas	[101,102]
Dermatophytosis (inflammatory form)	Fungal infection (e.g., *Microsporum canis*), inducing folliculitis and epidermal inflammation	Circular alopecia, scaling, erythema, mild pruritus; zoonotic potential	[98,103]
Otitis externa (inflammatory form)	Local inflammation of the external ear canal due to allergies, overgrowth of bacteria/yeasts (e.g., *Malassezia*, *Pseudomonas*), or foreign bodies	Ear scratching, head shaking, erythema, discharge, malodor, pain; often chronic and recurrent	[104,105,106]
Anal sacculitis	Inflammation of anal sacs due to impaction, infection, or allergy; mixed bacterial flora is often involved	Scooting, licking, painful defecation, swelling near the anus, purulent or bloody discharge	[107,108]
Localised post-surgical inflammation or trauma	Sterile or low-grade infectious inflammation due to surgical wounds, trauma, or abrasions	Erythema, oedema, mild exudate or crusts at wound margins; discomfort or pruritus	[109]
Pododermatitis (interdigital furunculosis)	Chronic inflammation often secondary to allergies, infection, foreign bodies, or conformation	Swelling, erythema, nodules, draining tracts between toes; painful walking	[110]
Mastitis (subclinical or clinical, in nursing bitches)	Inflammation of the mammary gland; bacterial (e.g., *E. coli*, *Staphylococcus* spp.), trauma or galactostasis	Local swelling, heat, pain, milk discolouration; systemic signs if severe	[111]

**Table 2:** Common inflammatory diseases in cats.

Disease	Pathophysiological Mechanism	Clinical Outcomes	References
Feline atopic skin syndrome	IgE and non-IgE-mediated hypersensitivity to environmental allergens; barrier dysfunction; Th2-type inflammation	Pruritus, facial and neck excoriations, miliary dermatitis, eosinophilic lesions, overgrooming, self-induced alopecia	[98]
Flea allergy dermatitis	Type I and IV hypersensitivity to flea saliva antigens	Intense pruritus, miliary dermatitis, crusted papules, alopecia, scabs, especially over the head, neck, and back	[115]
Eosinophilic granuloma complex	Eosinophil-dominated inflammatory response; hypersensitivity or idiopathic aetiology	Indolent ulcers, eosinophilic plaques (abdomen, thighs) and granulomas (limbs, chin)	[116,117]
Miliary dermatitis	Cutaneous reaction pattern, often triggered by food allergy or ectoparasites (e.g., *Notoedres cati*, *Cheyletiella*)	Small crusted papules, mainly on the dorsum, often accompanied by pruritus, alopecia	[118]
Feline acne (secondary folliculitis)	Follicular plugging and secondary inflammation; bacterial colonisation (e.g., *Staphylococcus*)	Chin and lower lip comedones, furunculosis, pustules, crusts, and swelling	[95,119]
Pemphigus foliaceus	Autoantibody-mediated targeting of desmosomal proteins (desmoglein-1); loss of keratinocyte adhesion	Crusting, pustules on face, pinnae, nasal planum, and feet; symmetrical or multifocal lesions	[120,121]
*Malassezia* dermatitis	Overgrowth of *Malassezia pachydermatis*, especially in allergic or immunocompromised cats	Greasy, erythematous, pruritic skin, particularly in intertriginous zones (axillae, neck, ear canal)	[99,100,122]
Bacterial pyoderma (uncommon)	Opportunistic bacterial infection (e.g., *Staphylococcus*); often secondary to allergies or ectoparasites (e.g., *Ctenocephalides felis*)	Pustules, papules, crusting, scab-like bumps; usually localised and chronic; less frequent than in dogs	[122,123]
Fungal dermatitis (e.g., dermatophytosis)	Infection by *Microsporum canis* or *Trichophyton* spp. causing folliculitis and inflammation	Circular alopecia, scaling, crusts, minimal pruritus; zoonotic; common in kittens and shelters	[103,110,124]
Allergic contact dermatitis	Type IV delayed hypersensitivity to topical agents (e.g., plastics, cleaners, ointments)	Erythema, vesicles, ulcers, pruritus; typically on sparsely haired areas in contact with allergen	[92,93]
Otitis externa (inflammatory or infectious)	Common in cats with allergies, polyps, or overgrowth of *Malassezia*, *Otodectes* spp., *Staphylococcus* or *Pseudomonas*; often treated with topical otic preparations	Otic inflammation is frequently associated with FASS or parasitic infestation	[125,126]
Parasitic dermatitis (e.g., *Notoedres cati*)	Highly pruritic, inflammatory skin reaction caused by mite infestation	Topical parasiticides (selamectin, moxidectin) are first-line therapy	[127,128]
Injection-site granulomas or inflammation	Post-vaccinal or injection-induced localised inflammation; important due to the risk of fibrosarcoma development	Treated with anti-inflammatory topicals or monitored; relevant in the pharmaceutical formulation context	[129]

**Table 3:** Common inflammatory diseases in farm animals.

Species	Disease	Pathophysiological Mechanism	Clinical Outcomes	References
Cattle	Dermatophilosis (rain scald)	*Dermatophilus congolensis* infection; moisture-induced maceration facilitates invasion of epidermis	Crusty, exudative dermatitis along the back and neck; matting of hair, pain, secondary bacterial infection	[131]
	Photosensitization (hepatogenous or primary)	Accumulation of photodynamic compounds (e.g., phylloerythrin); UV-induced skin inflammation	Erythema, oedema, necrosis of non-pigmented areas; severe pain, sloughing	[132]
	Papillomatosis (bovine warts)	Bovine Papillomavirus-induced epidermal hyperplasia; local inflammation	Firm, exophytic warts on head, neck, teats and udder; potential interference with milking or vision	[133,134]
	Digital dermatitis	*Treponema* spp. infection	Ulceration, crusts, and inflammation cause lameness	[135]
Sheep	Contagious ecthyma	Parapoxvirus infection; epidermal replication, cytolysis, and inflammation	Proliferative, crusting lesions around lips, nostrils, teats; painful, self-limiting, but zoonotic	[136,137]
	Fly strike (*Myiasis*)	Infestation with dipteran larvae (e.g., *Lucilia sericata*); necrotising inflammation due to tissue digestion	Moist, foul-smelling wounds; intense inflammation, pain, systemic toxaemia if severe	[138,139]
	Photosensitization	Same as in cattle (hepatogenous or primary); often due to *Lantana* ingestion or hepatic damage	Erythema, oedema, crusting and necrosis of exposed skin; reluctance to move	[132]
	Lice or mite infestation	Irritation from ectoparasite (e.g., *Bovicola ovi*, *Linognathus*, *Haematopinus* spp. feeding, mechanical injury, and hypersensitivity reactions	Pruritus, alopecia, crusts, thickened skin, secondary pyoderma	[140]
Goats	Caprine dermatophilosis	Similar pathogenesis to cattle, caused by the bacterium *Dermatophilus congolensis*; higher prevalence in humid climates	Crusts, alopecia on dorsal areas; lesions often painful	[141]
	Orf (contagious ecthyma)	As in sheep, infects the mucocutaneous junctions	Papules, vesicles, progressing to pustules and thick crusts, especially around the lips and nostrils	[136,142]
	Demodicosis (goat *Demodex* spp.)	Overgrowth of follicular *Demodex mites*; immune compromise may predispose	Nodular or diffuse dermatitis; alopecia, pruritus, thickened skin	[143,144]
Pigs	Exudative epidermitis (greasy pig disease)	*Staphylococcus hyicus* infection; exfoliative toxins cause epidermal damage and inflammation	Greasy exudation, crusting, especially in piglets; systemic signs may occur in severe cases	[145,146]
	Sarcoptic mange	Infestation with *Sarcoptes scabiei* var. *suis*; burrowing and hypersensitivity	Intense pruritus, erythema, crusts on ears, neck, and legs; weight loss in chronic cases	[147]
	Swine pox virus	Swinepox virus infection; viral replication in keratinocytes with inflammation	Papules, pustules, and scabs; typically mild, seen in young pigs	[148,149]
	Sunburn	UV-induced inflammation in non-pigmented or sparsely haired areas	Erythema, oedema, desquamation; behavioural signs of pain	[150]

**Table 4:** Emerging active pharmaceutical ingredients in topical veterinary anti-inflammatory therapy.

Therapeutic Class	Primary Mechanism of Action	Strategies	Clinical Implications and Limitations	References
Optimised topical corticosteroids (e.g., hydrocortisone aceponate)	Glucocorticoid receptor activation → NF-κB inhibition → ↓ IL-1β, TNF-α, IL-6	Lipophilic ester prodrugs; local bioactivation by esterases; vehicles favour epidermal retention	High local efficacy; ↓ HPA suppression vs. classical steroids; risk of atrophy if overused; ingestion risk in cats	[186,196]
Topical NSAIDs (COX-2 selective)	COX-2 inhibition → ↓ prostaglandins	Ionic pairing; supersaturation systems; nanocrystals to enhance dermal flux; reduce systemic exposure	Useful in musculoskeletal inflammation (dogs, horses); residue risk in food animals; limited in deep/systemic disease	[197,198]
Calcineurin inhibitors (e.g., tacrolimus)	Calcineurin blockade → ↓ NFAT activation → ↓ IL-2 transcription → T-cell suppression	Lipophilic ointments; permeability-enhancing vehicles	Alternative to steroids in atopic dermatitis; minimal atrophy; limited veterinary approval; species metabolic differences	[199]
JAK inhibitors (e.g., tofacitinib, oclacitinib)	JAK-STAT pathway inhibition → ↓ IL-31, IL-4, IL-13 signalling	Targeted cytokine modulation: adaptation from human dermatology	Promising in pruritic/Th2 conditions; need species-specific safety data; limited topical veterinary evidence	[200,201]
Specialised pro-resolving mediators (lipoxins, resolvins, protectins)	Promote resolution phase: ↑ efferocytosis; ↓ neutrophil infiltration	Encapsulation in liposomes, NLCs, and thermogels to prevent oxidation	Non-immunosuppressive; suitable for chronic inflammation; stability issues; limited clinical data	[202,203]
Antimicrobial and anti-inflammatory peptides (cathelicidins, defensins)	Bacterial membrane disruption; ↓ pro-inflammatory cytokines	Nanoemulsions; PLGA nanoparticles; cross-linked hydrogels for proteolytic protection	Potential reduction in antibiotic use, instability, high production cost, and regulatory uncertainty	[204,205,206]
Barrier-replenishing lipids (ceramides, cholesterol, phytosphingosine)	Restoration of stratum corneum lipid matrix; ↓ TEWL; ↓ antigen penetration	Lamellar emulsions; biomimetic nanostructures	Address pathophysiology of atopic dermatitis; minimal residue risk; supportive, not sufficient alone in severe cases	[207,208,209]
Natural bioactive compounds (polyphenols, flavonoids, algal polysaccharides)	NF-κB modulation; ROS scavenging; ↓ COX-2/iNOS expression	Green extraction (UAE, supercritical CO_2_); circular economy sourcing	Useful in mild/moderate inflammation; favourable ecotoxicology; variable potency; limited standardisation	[210,211,212]
Synthetic–natural co-formulations (e.g., NSAID + phenolic antioxidant)	Complementary cytokine inhibition; oxidative stress reduction	Dose-reduction strategy; antioxidant stabilisation; barrier support	Improved tolerability; potential synergism; requires mechanistic validation	[213,214]

**Table 5:** Emerging pharmaceutical formulations and drug delivery systems for topical veterinary anti-inflammatory therapy.

Type	Pharmacotechnical Characteristics	Advantages from the One Health Perspective	Limitations	Reference
Hydrogels (containing carbopol, HPMC, sodium hyaluronate)	Aqueous, biocompatible, non-greasy systems provide constant hydration and local cooling. Easy to prepare, suitable for water-soluble substances.	No organic solvents; completely biodegradable; safe for the operator and the environment; does not generate ecotoxic residues.	Requires preservatives (microbiological risk); low permeability for lipophilic molecules; reduced stability to contamination.	[268,269,270]
Organogels (lecithin/PMMA or PIM)	Lipophilic systems without water, suitable for insoluble compounds; they can form stable networks through self-association.	Eliminates the need for preservatives; good penetration for lipophilic molecules; lecithin is natural and biodegradable.	PIM and other synthetic esters may be poorly biodegradable, have a moderate risk of ecological accumulation, and have limited volatility.	[189,271,272]
Micro/nanoemulsions	Stable colloidal dispersions increase skin permeability; they can solubilise hydrophobic compounds.	Lower API doses required; may reduce systemic exposure; compatible with vegetable oils.	Requires surfactants and co-surfactants (sometimes irritants and non-biodegradable); ecotoxic impact if not reformulated “green.”	[273,274]
Liposomes, niosomes, transfersomes, ethosomes	Phospholipid or nonionic vesicles; controlled release; good skin retention.	Fully biodegradable; reduces active dose and risk of drug residues; minimises operator exposure.	Physicochemical instability (oxidation, hydrolysis); preservatives required; ethosomes may contain volatile ethanol.	[275,276,277,278]
Solid lipid systems	Solid lipid matrix; slow release; high physical stability; suitable for lipophilic substances.	No organic solvents; reduce application frequency; biocompatible; minimal environmental impact.	High-energy consumption production may require synthetic surfactants (Tween, Poloxamer).	[279,280]
Polymeric particles	Controlled release: reduces the need for frequent applications; protects sensitive molecules.	PLGA is completely biodegradable; metabolites (lactic/glycolic acid) are non-toxic; reduces exposure and contamination.	High cost; some preparation methods involve organic solvents (acetone, ethyl acetate).	[281,282]
Film-forming solutions	Solutions that form a protective film upon application, excellent dose control, and reduce the frequency of administration.	Minimises wash-off, reduces residue, compatible with biodegradable polymers, easy to use.	Some contain volatile organic compounds solvents, or non-biodegradable plasticisers; they require a balance between adhesion and permeability.	[283,284]
In situ gelatizable systems (poloxamer 407 thermogels, pH gels)	Gels at the application site (ear, wound); form a local deposit with controlled release.	Single administration, minimal environmental losses; low occupational exposure; high local retention.	Poloxamers and some synthetic polymers have limited biodegradability; pH adjustment may affect the local microbiome.	[285,286,287]

## Data Availability

No new data were created or analyzed in this study. Data sharing is not applicable to this article.
